# Phospho-Tau and Cognitive Decline in Alzheimer's Disease. Commentary: Tau in physiology and pathology

**DOI:** 10.3389/fnana.2016.00044

**Published:** 2016-04-28

**Authors:** Javier DeFelipe

**Affiliations:** Laboratorio Cajal de Circuitos Corticales (Centro de Tecnología Biomédica: UPM), CIBERNED, and Instituto Cajal (Consejo Superior de Investigaciones Científicas)Madrid, Spain

**Keywords:** Alzheimer's disease, human hippocampus and parahippocampal cortex, intracellular injections, pyramidal cells, dendritic spines, connections

In the brain, abnormal phosphorylation of tau leads to the formation of paired helical filaments, which are the main components of intraneuronal neurofibrillary tangles (NFT) (Grundke-Iqbal et al., 1986) that are characteristic of Alzheimer's disease (AD) and other tauopathies (Lee et al., 2001). There is an intense debate about the relationship of phospho-tau and the typical cognitive deficits in AD (Castellani et al., 2008) but, owing to the limited data that is available about synaptic circuits in the normal human brain and in that of the AD patient, the basic mechanism/s of cognitive deterioration are still a mystery. The article by Wang and Mandelkow (2016) presents a timely review of the roles of tau in physiology and disease. However, the role of hyperphosphorylation of tau in dendritic pathology is not fully addressed. Wang and Mandelkow concluded that NFT are probably unrelated to cognitive impairment, mainly based on studies using several lines of mice transgenic for wild-type or mutant human tau. However, in a previous study assessing the possible alterations to dendritic spines in pyramidal cells from AD patients (Merino-Serrais et al., 2013), we found a remarkable loss of dendritic spines from pyramidal cells containing NFT. Since pyramidal neurons represent the principal building blocks of the cerebral cortex and dendritic spines are the main postsynaptic elements of cortical excitatory synapses and are fundamental structures in memory, learning, and cognition (DeFelipe, 2015), these alterations constitute what we think is an important early event in the pathogenesis of AD. We used intracellular injections of Lucifer yellow (LY) in fixed brain tissue from AD patients to visualize and reconstruct dendritic arbors of pyramidal neurons—using high-resolution tile scan stacks of confocal microscopy images—to compare neurons that were free of signs of any neurofibrillary pathology with those showing either diffuse phospho-tau (putative pre-tangle state) or aggregated tau NFT.

Following injection in the parahippocampal cortex and CA1, the sections were immunostained for LY and phospho-tau. In the so-called putative “pre-tangle” stage, the dendritic trees of pyramidal neurons were unchanged (pattern I; Figure 1, Panel 1). In the presence of well-developed NFT, however, dendritic spine loss was obvious (pattern IIb; Figure 1, Panel 2). In cases with an intermediate state of neurofibrillary pathology (pattern IIa), the loss of dendritic spines was more variable. Importantly, we compared neighboring cells with and without neurofibrillary pathology (see A, B in Figure 1, Panel 1) to avoid confounding factors such as: (1) morphological differences in the structure of pyramidal cells due to regional specializations (i.e., pyramidal cells in different cortical regions and layers may show morphological differences); (2) high inter-individual variability (sex, age, medical treatment, etc.,) factors that could affect brain structure; and (3) the highly variable course of AD, as the neuropathological changes are not homogenous among patients or in different regions of the brain of the same patient, giving rise to variation in the alterations to cortical circuits.

**Figure 1:**
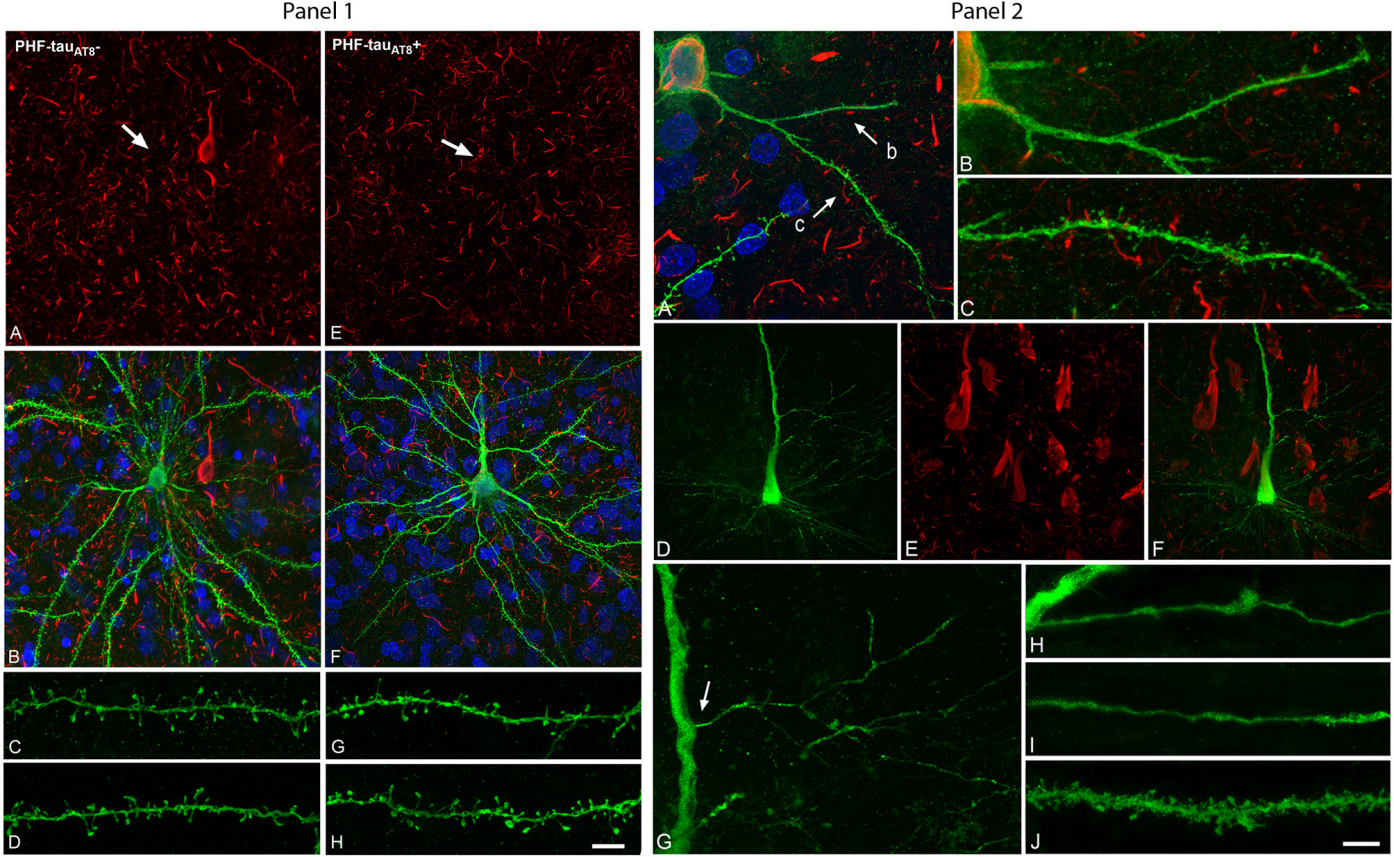
**Panel 1**. Neurons and dendrites in the parahippocampal cortex of patient P9 injected with LY whose soma is free of phospho-tau PHF-tau_AT8_-ir (${\text{PHF-tau}}_{AT8}^{-}$; **A–D)** or contains phospho-tau ${\text{(PHF-tau}}_{AT8}^{+}$ in the putative pre-tangle state (Pattern I; **E–H)**. Stacks of 26 **(A**,**B)** and 28 **(E,F)** confocal optical sections, respectively, obtained after combining the channels acquired separately for DAPI (blue), LY (green), and PHF-tau_AT8_ immunostaining (red). Stacks of 26 **(C,D)** and (32 **(G,H)** confocal optical sections, respectively, from basal dendrites of ${\text{PHF-tau}}_{AT8}^{-}$ **(C,D)** and immunostained ${\text{(PHF-tau}}_{AT8}^{+}$; **G,H)** LY-injected pyramidal neurons_._ No changes were found in dendrite diameter or in the number, length or volume of the dendritic spines in neurons with Pattern I. Arrows in **(A)** and **(E)** indicate the localization of the cell body of the LY-injected pyramidal neurons shown in **(B)** and **(F)**, respectively. Scale bar in **(H)**: 13 μm in **(A,B,E**,**F)**; 2 μm in **(C,D,G**,**H)**. Taken from Merino-Serrais et al. (2013). **Panel 2**. Alterations of dendrites and dendritic spines in LY-injected neurons with extensive neurofibrillary tangles (Pattern IIb of immunostaining), in the parahippocampal cortex in patient P9 **(A–C)** and in the CA1 of patient P12 **(D–J)** that were PHF-tau_AT8_-ir or PHF-tau_*PHF*1_-ir, respectively. **(A)**, Stack of 27 confocal optical sections obtained after combining the channels acquired separately for DAPI (blue), LY (green), and PHF-tau_AT8_-ir (red), showing the cell body and proximal dendrites of the intracellularly labeled neuron that contains phospho-tau. **(B,C)**, Higher magnification of **(A)**, showing the dendrites indicated as b and c, respectively. Note the low density of dendritic spines in dendrite b compared to dendrite c, as well as the low spine density in dendrite c compared to the dendrites in **Panel 1** from neurons that are free of phospho-tau or that contain phospho-tau in the putative pre-tangle state. The dendritic spines are also notably smaller. **(D–E)** Stacks of 27 confocal optical sections showing the cell body and proximal dendrites of the intracellularly labeled neuron **(D)** that contains phospho-tau **(E)**. **(F)** Image obtained by combining panels **(D)** and **(E)**. **(G)** Higher magnification of **(D)**. **(H**,**I)** Stacks of 38–55 confocal optical sections from a collateral apical dendrite (arrow in **G)** of the LY-injected pyramidal neuron, showing different segments of the same dendrite **(H**, proximal; **I**, distant). **(J)** Stack of 26 confocal optical sections from the collateral apical dendrite of an intracellularly labeled neuron that was adjacent to the LY-injected neuron shown in **(D)** and that was free of phospho-tau. Note the lack of dendritic spines and the thin diameter of the dendrites of the neurons containing phospho-tau **(H,I)** compared to the dendrite of the neuron that was free of phospho-tau **(J)**. Scale bar in **(J)**: 10 μm in **(A);** 3.5 μm in **(B,C)**; 20 μm in **(D–F)**; 9 μm in **(G)**; 4.5 in **(H–J)**. Taken from Merino-Serrais et al. (2013).

We concluded that the presence of phospho-tau in neurons does not necessarily mean that they suffer severe and irreversible effects as thought previously, but rather the characteristic cognitive impairment in AD is likely to depend on the relative number of neurons that have well-developed tangles. Certainly, these observations differ from those of studies on transgenic mouse brains, where human mutant tau was overexpressed. However, this discrepancy could be explained by the fact that transgenic mouse brains do not reproduce all features of AD found in humans. Furthermore, there are many molecular and structural differences between mouse and human brain. Thus, extrapolation of data obtained in mice and humans is rather difficult and possible mismatches between the two species should be taken into consideration when dealing with animal models of AD.

## Author contributions

The author confirms being the sole contributor of this work and approved it for publication.

### Conflict of interest statement

The author declares that the research was conducted in the absence of any commercial or financial relationships that could be construed as a potential conflict of interest.
